# Supplementary material for: The Multicellular Effects of VDAC1 N-Terminal-Derived Peptide
Source: Biomolecules. 2022 Sep 28;12(10):1387. doi: 10.3390/biom12101387 (PMC9599717; doi:10.3390/biom12101387)
Supplement: Supplementary file 1 [file biomolecules-12-01387-s001.zip › supplementary Tables and Figures.pdf]

Supplementary Data

The multicellular effects of VDAC1 N-terminal-derived peptide

Uttpal Anand[1], Anna Shteinfer-Kuzmine[2], Gal Sela[1], Manikandan Santhanam[1], Benjamin Gottschalk[3], Swaroop Pandey[2], Boujemaa-Paterski[4], Ohad Medalia[4,] Wolfgang F. Graier[3,5] and Varda Shoshan-Barmaz[1,2]#

[1]Department of Life Sciences , Ben-Gurion University of the Negev, Beer-Sheva 84105, Israel [2]National Institute for Biotechnology in the Negev, Ben-Gurion University of the Negev, Beer-Sheva 84105, Israel, [3]Gottfried Schatz Research Center: Molecular Biology and Biochemistry, Medical University of Graz, Austria, [4]Department of Biochemistry, University of Zurich, 8057 Zurich, Switzerland, [5]BioTechMed Graz, Austria

Materials

Acridine orange/ethidium bromide (AO/EtBr), bovine serum albumin (BSA), Dimethyl sulfoxide (DMSO), 4′,6-diamidino-2-phenylindole (DAPI), leupeptin, phenylmethylsulfonyl fluoride (PMSF), fluorescein isothiocyanate (FITC), propidium iodide (PI), Triton X-100, Tween-20, and trypan blue were purchased from Sigma (St. Louis, MO, USA). Annexin V (FITC) was obtained from Alexis Biochemicals (Lausen, Switzerland). Eagle's Minimum Essential Medium (EMEM), Dulbecco’s Modified Eagle’s Medium (DMEM), and Dulbecco’s phosphate-buffered saline (DPBS) were obtained from Gibco (Grand Island, NY, USA). Normal goat serum (NGS) and the supplement fetal bovine serum (FBS), non-essential amino acids (NEAA), sodium pyruvate, L-glutamine, and penicillin-streptomycin were obtained from Biological Industries (Beit-Haemek, Israel). Sodium dodecyl sulfate 20% was purchased from Bio-Lab Ltd. (Jerusalem, Israel). *N*-(3-Triethylammoniumpropyl)-4-(6-(4-(Diethylamino) Phenyl) Hexatrienyl) Pyridinium Dibromide (FM4-64 dye), and Alexa-fluor 488-Phalloidin were from ThermoFisher Scientific (Waltham, MA, USA). Primary antibodies, their sources, and the dilutions used are detailed in Table 1. Horseradish peroxidase (HRP)-conjugated, and fluorophore-conjugated secondary antibodies are also listed in Table S1.

Methods

Lipid staining with Oil Red O

Cells were treated with various concentrations of D-Δ(1-18)N-Ter-Antp peptide in serum-free DMEM media for 6, 12, and 24 h. Following this, media were removed from the culture plate, and cells were washed with PBS, fixed with formalin (10%) for 30 min at room temperature (RT), and then washed with double distilled water (DDW). Isopropanol (60%) was added to each well and incubated for 5 min at RT. After incubation, the isopropanol was removed from the well, and Oil Red O working staining solution [Oil Red O: distilled water (3:1)] was added after filtration with a 0.2-μm syringe filter to each well. The plates were slowly rotated and incubated for 20 min at RT. DDW was applied to the plate to remove the excess Oil Red O staining solution. Cells were kept in DDW during imaging.

Oil Red O cell staining was measured by extraction with isopropanol (100%) after washing and incubated for 5 min with gentle rocking. Absorbance was read at 492 nm.

Cell glycogen level determination

U-87MG cells were treated with various concentrations of D-Δ(1-18)N-Ter-Antp peptide in serum-free DMEM media for 24 h. The glycogen content was measured using the anthrone reagent, as described previously with minor modification [30]. Cells were harvested, re-suspended in KOH (30%), and maintained in a boiling water bath (100°C) for 30 min. Glycogen was precipitated by adding $NaSO_4$ (1M) and ethanol. The mixture was thoroughly mixed and boiled in a water bath. A glycogen rich pellet was obtained by centrifugation (10,000 g, 5 min). Supernatant was discarded, and the pellet was dissolved in DWW, followed by a second and third precipitation carried out as above. The precipitate was re-suspended in DDW and anthrone (0.2%) in 95% sulfuric acid under ice-cold conditions, heated in a boiling water bath for 10 min, cooled immediately, and the

color was read in a spectrophotometer at 680 nm. A standard curve was obtained using D-glucose (0–20 μg) subjected to anthrone in a sulfuric acid treatment, as described above.

Lysosomal vacuolation observed with acridine orange (AO) staining

U-87MG cells were treated with D-Δ(1-18)N-Ter-Antp peptide in serum-free DMEM media for 6 h at 37°C, incubated with acridine orange (1 μg/ml) for 15 min, visualized by fluorescence microscopy (Olympus LX2-KSP, Tokyo, Japan), and images were captured with a CCD camera. About 200 cells were counted for each experiment.

Cell migration assay/wound-healing assay

U-87MG cells were seeded in 12-well plates and cultured. When reaching around 90% confluence, a straight scratch was mechanically made with a sterile 10-μl pipette tip. The cells were treated with various concentrations of D-Δ(1-18)N-Ter-Antp peptide in serum-free DMEM media for the indicated time points. The images of the wounds were captured at different time points using a digital camera mounted on a microscope to follow the position of the migrating front at defined times.

Cell senescence assay

U-87 MG cells were treated with the indicated concentrations of D-Δ(1-18)N-Ter-Antp peptide for 6 h, then the medium containing peptide was removed, and cells were washed with PBS. The cells were fixed with 0.5% glutaraldehyde solution (in PBS pH 7.4) for 15 min at RT and washed with PBS for 5 min. Then cells were washed with PBS/$MgCl_2$ (pH 6) for 5 min at RT. X-Gal staining solution (0.2M $K_3Fe(CN)_6$, 0.2M $K_4Fe(CN)_6$ $3H_2O$, X-Gal in PBS/$MgCl_2$) was freshly prepared and added to the cells. The cells were incubated at 37°C for 5 h, sealed and protected from light. They were then washed with pre-warmed PBS (37–55°C) for 5 min at RT, visualized for SA-β-gal by an Olympus (LX2-KSP, Tokyo, Japan) microscope, and images were captured with a CCD camera.

Cell penetration of fluorescein isothiocyanate (FITC)-labeled VDAC1-derived D-Δ(1-18)N-Ter-Antp peptide

To fluorescently label the D-Δ(1-18)N-Ter-Antp-peptide, it was incubated with FITC (50 μM) for 30 min in Tricine buffer (10 mM, pH 8.7), at 37°C in the dark. Unreacted reagent was removed by dialysis using membranes with a cut-off of 1,000 Da (DiaEASY Dialyzer Floating Rack; BioVision). U-87MG cells were seeded on sterilized coverslips and were incubated with the indicated concentrations of FITC-labeled peptide in serum-free DMEM for 2 h, washed with PBS, fixed with 4% paraformaldehyde for 20 min and washed three times with PBS, permeabilized with PBST, and blocked with blocking buffer for 2 h. Primary antibody was probed in an antibody buffer overnight at 4°C. The next day, cells were washed three times with PBS and probed with fluorophore-conjugated secondary antibody for 2 h at RT. Following secondary antibody incubation, coverslips were washed three times with PBS and incubated with DAPI for 15 min in the dark, and carefully washed, dried, and mounted on slides using fluoroshield mounting medium. After overnight drying, images were acquired using an Olympus IX81 confocal microscope.

Table S1. **Antibodies used in this study**

Antibodies against the indicated protein, their catalogue number, source, and the dilutions used in immunoblotting (WB) and immunofluorescence (IF) experiments are presented below.

Antibody	Source and catalog number	Dilution	
		**WB**	**IF**
Mouse monoclonal anti-actin	Millipore, Billerica, MA, MAB1501	1:40,000	-
Goat anti-Mouse-HRP	Abcam, Cambridge, UK, ab97040	1:10,000	-
Goat anti-Rabbit- HRP	Promega, Madison, WI, W4011	1:15,000	-
Goat anti-Rabbit H&L-Alexa Fluor 555	Abcam, Cambridge, UK, ab150078	-	1:750
Goat anti-Rabbit H&L-Alexa Fluor 488	Abcam, Cambridge, UK, ab150077	-	1:750
Goat anti-Mouse H&L-Alexa Fluor 555	Abcam, Cambridge, UK, ab150113	-	1:750
Goat anti-Mouse H&L-Alexa Fluor 488	Abcam, Cambridge, UK, ab150114	-	1:750
Rabbit monoclonal anti-AIF	Abcam, Cambridge, UK, ab32516	1:2000	
Rabbit polyclonal anti-ATP synthase 5a	Abcam, Cambridge, UK, ab151229	1:2000	-
Rabbit polyclonal anti-citrate synthetase	Abcam, Cambridge, UK, ab96600	1:2000	-
Rabbit monoclonal anti-CDK2	Abcam, Cambridge, UK, Ab32147	1:2000	-
Rabbit monoclonal anti-IκB-α	Abcam, Cambridge, UK, Ab32518	1:2000	-
Rabbit monoclonal anti-hexokinase I	Abcam, Cambridge, UK, ab150423	1:2000	-
Rabbit polyclonal anti-VDAC1	Abcam, Cambridge, UK, ab15895	1:5000	1:500
Rabbit monoclonal anti-beta tubulin	Abcam, Cambridge, UK, ab179513	-	1:2000
Rabbit-monoclonal anti-vinculin	Abcam, Cambridge, UK, ab129002	-	1:2000
Rabbit monoclonal anti-BAX	Abcam, Cambridge, UK, ab32503	1:2000	-
Rabbit polyclonal anti-Bcl-xL	Abcam, Cambridge, UK, ab98143	1:1000	-
Rabbit polyclonal anti-Bcl-2	Abcam, Cambridge, UK, ab196495	1:2000	-
Rabbit polyclonal anti-p53	Abcam, Cambridge, UK, ab131442	1:2000	-
Rabbit polyclonal anti-c-Jun	Abcam, Cambridge, UK, ab31419	1:2000	-
Rabbit polyclonal anti-NF-κB p65	BioLegend, San Diego, CA, 622601	1:2000	-
Mouse monoclonal anti-caspase-9	Cell Signaling Technology, 9508S	1:1000	-
Rabbit polyclonal anti-LC3II	Sigma–Aldrich, St. Louis, MO, L7543	1:1000	-

Table S2. VDAC1-N-terminus-derived peptides

The bold letters indicate the cell-penetrating peptide sequence, and the underlined sequences represent amino acids in D conformation.

Peptide	Sequence	No. of AA	Molecular Mass, Da	Purity %
**Δ(1-14)N-Ter-Antp**	15-RDVFTKGYGFGL-26- **RQIKIWFQNRRMKWKK**	28	3588	95.73
**D-Δ(1-14)N-Ter-Antp**	15-RDVFTKGYGFGL-26- **RQIKIWFQNRRMKWKK**	28	3588	95.79
**N-Ter-Antp**	1-MAVPPTYADLGKSARDVFTKGYGFGL-26- **RQIKIWFQNRRMKWKK**	42	4990	97.72
**Δ(1-10)N-Ter-Antp**	11-GKSARDVFTKGYGFGL-26- **RQIKIWFQNRRMKWKK**	32	3932	86.59
**N-Ter Δ(21-26)-Antp**	1-**MAVPPTYADLGKSARDVFTK**-20- RQIKIWFQNRRMKWKK	36	4396	87.92
**D-Δ(1-18)N-Ter-Antp**	19-**FTKGYGFGL**-26- RQIKIWFQNRRMKWKK	25	3218	98.21
**Δ(1-10)N-Ter-min-Antp**	11-GKSARDVFTKGYGFGL-26- **RRMKWKK**	23	2718	95.24

Table S3. Summary of the results with various modified VDAC1-based peptides inducing cell death of cancer cell lines

Summary of the peptide concentrations (μM) required for half-maximal cell death activity (IC_{50}) in various cancer cell lines. Cells were incubated with different peptide concentrations for 1.5 h (MEC-1) or 6 h for the other cell lines. Cell death was determined by PI staining and FACS analysis. Minimal Antp represents the following sequence presented in Table S2.

Peptide	Cell line	IC_{50}, μM	Concentration for maximal cell death
**Δ(1-14)N-Ter-Antp**	PC-3<br>MEC-1	3.5<br>~6	12.5 μM, 80%<br>17 μM, 60%
**D-Δ(1-14)N-Ter-Antp**	PC-3<br>T-Rex-293<br>MEC-1	2<br>4<br>3	10 μM. 90%<br>15 μM, 35%<br>15 μM, 95%
**N-Ter-Antp**	MEC-1	5	12.5 μM, 80%
**Δ(1-10)N-Ter-Antp**	MEC-1	3	12.5 μM, 80%
**N-Ter Δ(21-26)-Antp**	MEC-1	>12.5	12.5 μM, 5%
**D-Δ(1-18)N-Ter-Antp**	U-87MG<br>HeLa	6<br>2	15 μM, 70%<br>15 μM, 100%
**Δ(1-10)N-Ter-min-Antp**	MEC-1	>15	15 μM, 20%

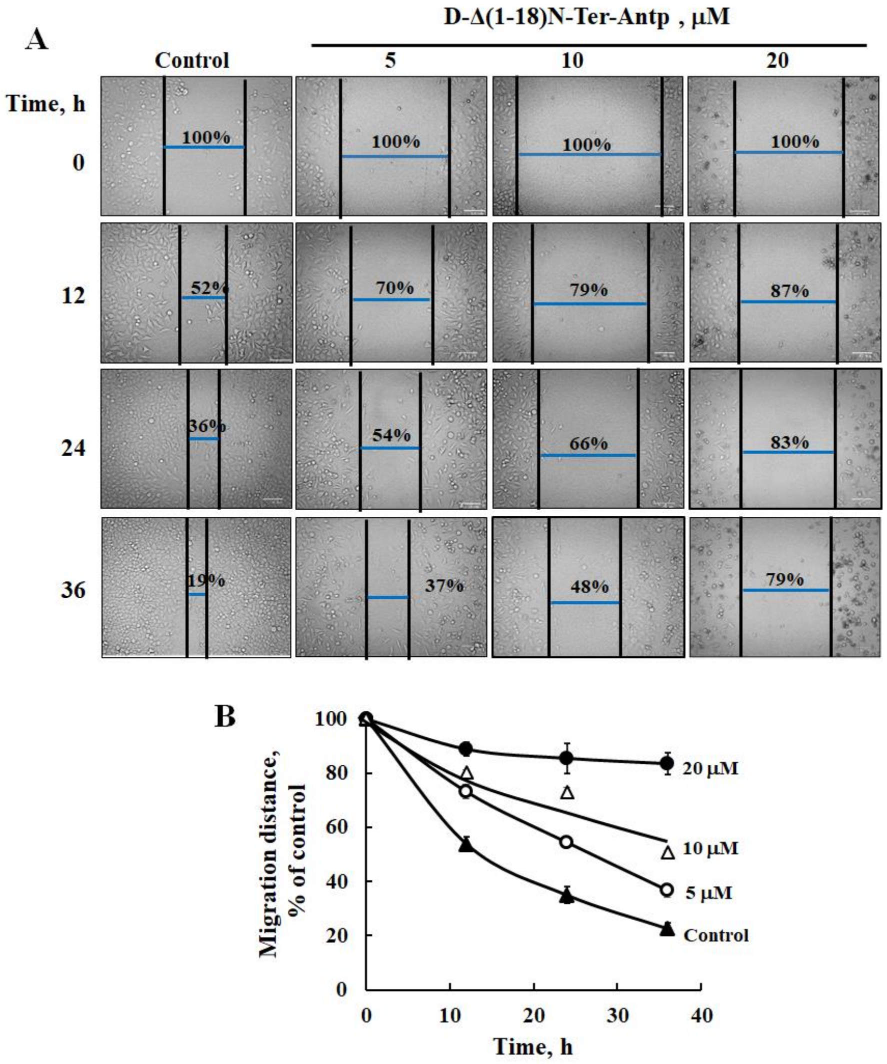

Figure S1. D-Δ(1-18)N-Ter-Antp peptide inhibits U-87MG cell migration. U-87MG cells were cultured in 12-well plates in DMEM containing 10% FBS. When cell confluent was 85–90%, a strip of cells was removed with a sterile 10-μl pipette tip. Cells were then incubated with the indicated concentrations of D-Δ(1-18)N-Ter-Antp peptide for 0, 12, 24, and 36 h, and gap closure during the stated time period was captured using a microscope equipped with CCD camera. **A**. Representative microscopic images of U-87MG cells treated or untreated with the peptide for the indicated concentration and time. **B**. Quantification of the results, shown as the mean ± SD, *n* = 3. The images were captured at 20X.

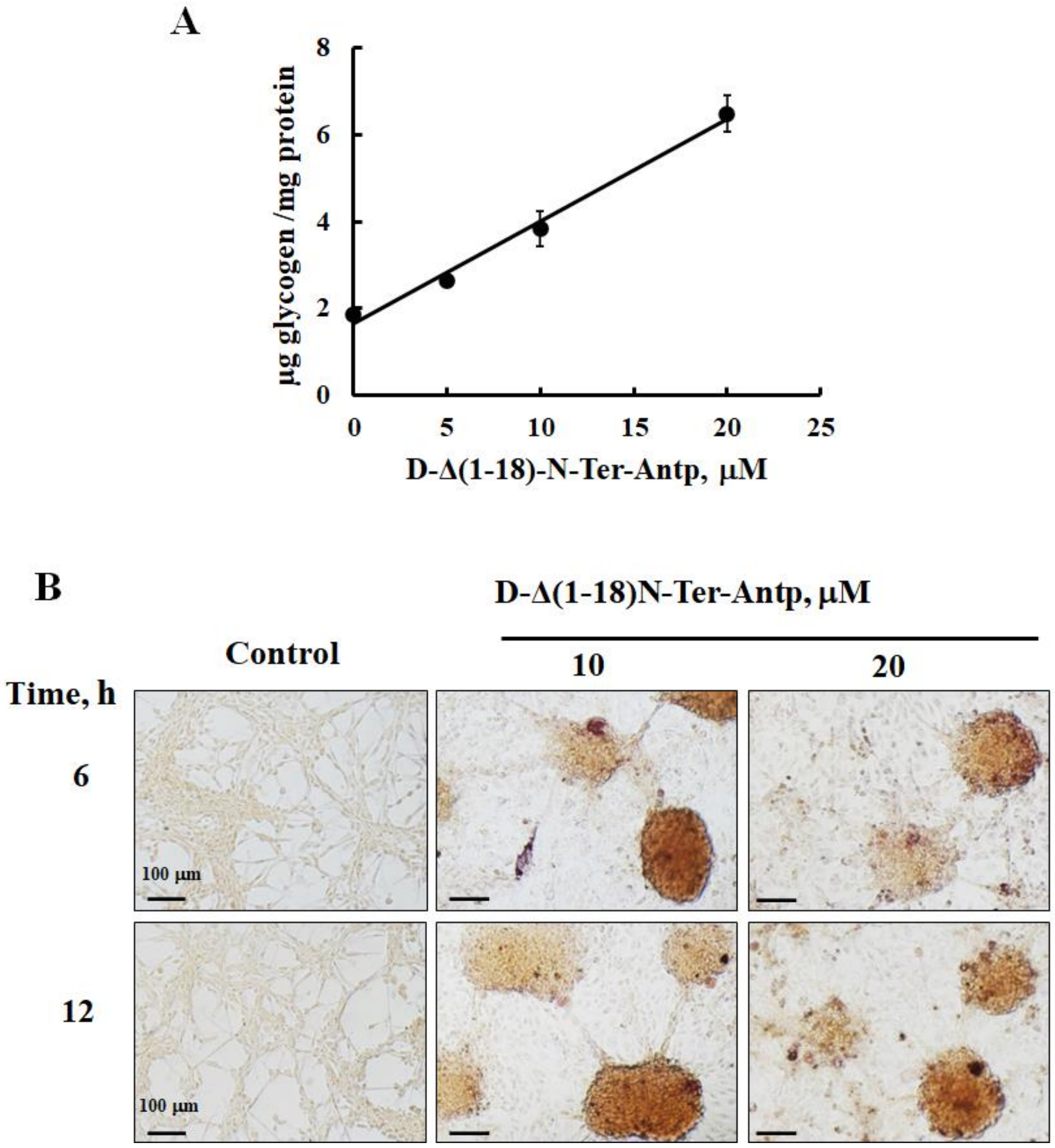

Figure S2. D-Δ(1-18)N-Ter-Antp peptide induced glycogen particles and lipids accumulation
A .U-87MG cells were treated with the indicated concentrations of D-Δ(1-18)N-Ter-Antp peptide for 16 h, and glycogen levels were determined as described in above. Results are presented as the mean ± SD, $n = 3$. **B. U**-87MG cells were treated with the indicated concentrations of D-Δ(1-18)N-Ter-Antp peptide for 6 or 12 h and were treated with Oil Red O to visualize accumulated lipids as described in the Methods section.
